# Biphasic Change of Tau (τ) in Mice as Arterial Load Acutely Increased with Phenylephrine Injection

**DOI:** 10.1371/journal.pone.0060580

**Published:** 2013-04-08

**Authors:** Bo Yang, Douglas F. Larson, James Ranger-Moore

**Affiliations:** 1 Department of Cardiac Surgery, Cardiovascular Center, University of Michigan, Ann Arbor, Michigan, United States of America; 2 Sarver Heart Center and the Department of Medical Pharmacology, College of Medicine, The University of Arizona, Tucson, Arizona, United States of America; 3 Division of Epidemiology and Biostatistics, Mel and Enid Zuckerman College of Public Health, The University of Arizona, Tucson, Arizona, United States of America; University of Illinois at Chicago, United States of America

## Abstract

**Background:**

Diastolic dysfunction is the hemodynamic hallmark of hypertensive heart disease. Tau (τ) has been used to describe left ventricle relaxation. The relationship between τ and afterload has been controversial. Our goal was to demonstrate this relationship in mice, because genetically-modified mouse models have been used extensively for studies in cardiovascular diseases.

**Methods:**

Increased arterial load was produced by phenylephrine administration (50 µg/kg iv) (n = 10). A series of pressure-volume loops was recorded with a Millar conductance catheter *in vivo* as the left ventricle pressure reached the maximum. The arterial load was expressed as Ea (effective arterial elastance). Tau values were computed using three mathematical methods: τ_Weiss_, τ_Glantz_, and τ_Logistic_.

**Results:**

A correlation plot between τ and Ea showed a biphasic relationship a flat phase I and an inclined phase II. The existence of an inflection point was proved mathematically with biphasic linear regression. Pressure-volume area (PVA), a parameter linearly related to myocardial O_2_ consumption (MVO_2_), was found to be directly proportional to Ea. The plot of τ versus PVA was also biphasic.

**Conclusion:**

We concluded that a small increase of the arterial load by phenylephrine increased PVA (index of MVO_2_) but had little effect on τ. However, after an inflection point, further increase of arterial load and PVA resulted in the linear increase of τ.

## Introduction

The cardiac cycle is divided into systole and diastole, where diastole can be further separated into two phases: isovolumic relaxation and passive filling. Diastolic dysfunction is a syndrome characterized by impaired ventricular filling resulting from prolonged left ventricular (LV) relaxation and/or increased LV stiffness. More than 4.6 million people in the United States have chronic heart failure, and at least one-third of these patients can be considered to have diastolic heart failure [1]. Compared to patients with systolic heart failure, patients with diastolic heart failure are more likely to have elevated blood pressure at the time of presentation and most patients have a history of hypertension [2]. Diastolic dysfunction is the hemodynamic hallmark of hypertensive heart disease.

There are two parameters used to describe the active isovolumic relaxation of LV in diastole: tau (τ) and dP/dt_min_. Tau is defined as a time constant of isovolumic relaxation and considered to be a more accurate parameter than dP/dt_min_ [3]. There are three computational methods used to measure τ. Tau was first used by Weiss to describe the isovolumic relaxation of LV [4]. The regression of a natural log of LV pressure against time in a monoexponential mode is linear, and 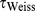 was originally defined as the negative inverse of the slope [4]. In order to eliminate the influence of non-zero asymptote, Raff and Glantz proposed an alternative method to compute tau, referred to as 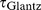. Mathematically, the regression of dP/dt against LV pressure is also linear in the same mono-exponential model, and 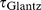 is equal to the negative inverse of the regression slope [5]. Because the mono-exponential model has a small deviation from linearity and may not be precise enough to characterize the LV isovolumic relaxation, Marsubara et al. proposed a third model: the logistic model,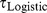. The advantage of the logistic model over the exponential model is that 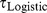 is insensitive to the choice of isovolumic relaxation cutoff point [6]. However, the exponential model still creates an acceptable approximation, and τ_Weiss_ appears to estimate the true τ [7].

Phenylephrine has been used to treat hypotension in clinic settings by causing peripheral vasoconstriction and increasing afterload. The direct effect of afterload on isovolumic relaxation and, therefore τ, is unclear. This issue becomes more complicated when studied *in vivo* in whole animals. Increasing afterload by mechanical aortic compression or administration of phenylephrine, methoxamine, or angiotensin II has been found to increase τ significantly [8], [9], [10], [11], moderately [12], or not at all [13]. Inter-species variability was also addressed by Leite-Moreira et al. as a confounding issue [14], [15]. In ferret [16], dog [17], and rabbit [18] hearts, a concomitant increase in τ was observed as afterload was increased under isovolumic controlled conditions. Minimal effects were detected in rat, guinea pig [12], rabbit [19], and human hearts [13]. Most of these studies measured 2–4 points, thus limiting their capability to describe the τ response in relationship to increased afterload. Furthermore, these studies computed τ using various methods, including mono-exponential and logistic models.

Lately, genetically-modified mouse models have been used to study the properties of LV relaxation, such as phospholamban knock-out mice, sarcoplasmic reticulum Ca^++^ ATPase (SERCA), and Na^+^ - Ca^++^ exchanger (NCX) transgenic mice. It is very important to characterize the relationship of τ and afterload in mice in order to understand those genetic modifications and the function of certain proteins related to the diastolic function. In the current study, we used a combined Pressure-Conductance Catheter System to generate pressure-volume loops and measure LV mechanics in mice. We applied all three computational methods described above for expressing τ. A series of pressure-volume loops was recorded to measure the change in τ as arterial load was acutely increased with phenylephrine administration. Our goal was to demonstrate how acutely increasing arterial load with phenylephrine affects cardiac diastolic function in mice and discuss the potential mechanisms.

## Methods

### Animals and Preparation

The University of Arizona Animal Review Committee approved these animal studies, which were in compliance with the “Guidelines for the Care and Use of Laboratory Animals” (NIH publication No. 86–23, revised 1985) and “Principles of Laboratory Animal Care” (published by the National Society for Medical Research). Male C57BL/6 mice, 6 months old, were obtained from the National Institute of Aging, Washington, DC. The animals were housed in the animal facility of the Arizona Health Sciences Center under diurnal lighting conditions and with unlimited access to food and water for two weeks before the study.

### Conductance Catheter System (CCS)

A combined catheter with four conductance electrodes and a micromanometer (Millar 1.4 F, SPR-716) was used for quantification of the pressure-volume relationships. The pressure transducer of the CCS was calibrated in saline maintained at 37°C and exposed to ambient atmospheric pressure. The Millar Pressure and Conductance System Controller (Millar MPCU-200) was set according to the manufacturer’s recommendations with an excitation frequency of 20 kHz and output filter frequency of 500 Hz. The signals were acquired at a rate of 1,000 samples/second (approximately 110 samples/cardiac cycles) with the custom software (BioBench, National Instruments, Austin, TX). Volume was calibrated with a volume calibration line (VCL) derived from a calibrator, and parallel volume (Vp) [20], [21], [22].

### In vivo Hemodynamic Measurement

The *in vivo* application of the Millar Conductance Catheter System was performed as described by Yang et al. [20], [21], [22]. After the induction of anesthesia with urethane (1000 mg/kg, ip) and α-chloralose (50 mg/kg, ip), the mice were ventilated through a tracheostomy with a pressure-controlled respirator (RSP 1002, Kent, CT) at a rate of 120 times/minute and FIO_2_ of 1.0. Normal saline and drugs were administered through the external jugular vein. A clamshell incision was made to expose the cardiac apex and inferior vena cava (IVC). Through an apical stab wound made with a 25-gauge needle, the Millar conductance catheter was inserted into the left ventricle and positioned along the cardiac longitudinal axis with the distal electrode in the aortic root and the proximal one in the cardiac apex. Ventilation was paused for 3–4 seconds, while pressure-volume relationships were acquired. The arterial load was modeled by infusion of a phenylephrine bolus iv (50 µg/kg, in less than 10 µl). Finally, a bolus of 15% saline (10 µl) was injected through the external jugular vein for the Vp calibration [22].

### Data Analysis

#### Calculation of τ

We used the customized software Pvan (Pvan version 2.9, Conductance Technologies Inc., San Antonio, TX, and Millar Inc., Houston, TX) to analyze the pressure-volume data exported from Biobench. Three τ’s (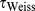, 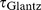, and 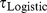) were computed using the diastolic portion of the LV pressure waveform starting at dP/dt minute. 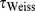 was computed using a mono-exponential model and a regression analysis of the natural log of LV pressure versus time [equation 1] as pressure decreased during diastole [4]. 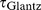 was computed from the same model as 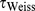, but using regression analysis of dP/dt versus LV pressure [equation 2] [5], [7], [23], and 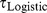 was computed from the logistic model [equation 3] [6].
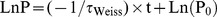
(1)

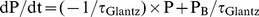
(2)

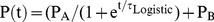
(3)P or P(t): LV pressure; P_0_ or P_A_: amplitude constant; P_B_: nonzero asymptote; and t: time.

#### Multi-regression of τ versus HR, Ped, and Ea

After computing τ, heart rate (HR), end diastolic pressure (Ped), and effective arterial elastance (Ea) using Pvan software, we applied a quadratic term to test the effects of Ea, Ped, and heart rate on τ during the acutely increasing arterial load by phenylephrine. Ea was used to represent the arterial load and calculated as end systolic pressure (Pes) divided by stroke volume (SV): Ea = Pes/SV [24], [25], [26]. Ped was used to represent preload. Since the relationship between τ and Ea was nonlinear, a quadratic term was included in the analysis. The multiple regression was conducted according to the following formula:
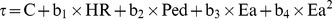
(4)where C is a constant and b_1_, b_2_, b_3_, and b_4_ are coefficients. Estimation of equation 4 by ordinary least squares showed b_1_ and b_2_ to be non-significant, and b_3_ and b_4_ to be significant with p<0.05. This suggested that the arterial load described by Ea independently caused a significant change in τ after intravenous injection of phenylepherine.

#### Identifying the inflection point

The plot of τ versus Ea showed a curve of biphasic pattern. Two separate single linear regressions were conducted for phases I and II respectively. The intersection point of these two linear regressions was calculated. The closest point to the intersection point was identified as the inflection point. (See Appendix S1 for more details regarding this calculation). Once the inflection point was identified, biphasic linear regression was applied to τ versus Ea, and τ versus PVA (pressure volume area).

#### Biphasic linear regression

After we identified the inflection point in the plot of τ versus Ea, we applied a biphasic linear regression analysis of τ versus Ea [equation 5] since the plot of τ versus Ea suggested a biphasic linear relationship:
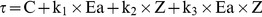
(5)where C is a constant and k_1_, k_2_, and k_3_ are coefficients. The indicator variable Z is set to 0 in phase I, and set to 1 in phase II, and serves to establish the interaction term that allows for the possibility of different slopes before and after the inflection point. If no such inflection point existed, the parameter estimate for the interaction term, k_3_, would not be statistically significant.

The quadratic model [equation 4] provided a mathematical rationale for selecting an inflection point at which the slope change occurred. The biphasic linear regression resulted in a significantly greater R^2^, supporting the choice of a biphasic linear model. (See Appendix S1 for details regarding sample analysis).

### Statistics

Statistical significance of coefficients in the regression models was determined using p-values derived from the student’s t-test associated with linear regression. Statistical significance for intra-group or inter-group was determined using p-values derived from the paired t-test or unpaired t-test. For all models, p<0.05 was considered to be statistically significant.

## Results

### Modulation of Afterload with Phenylephrine Administration

**Figure 1:**
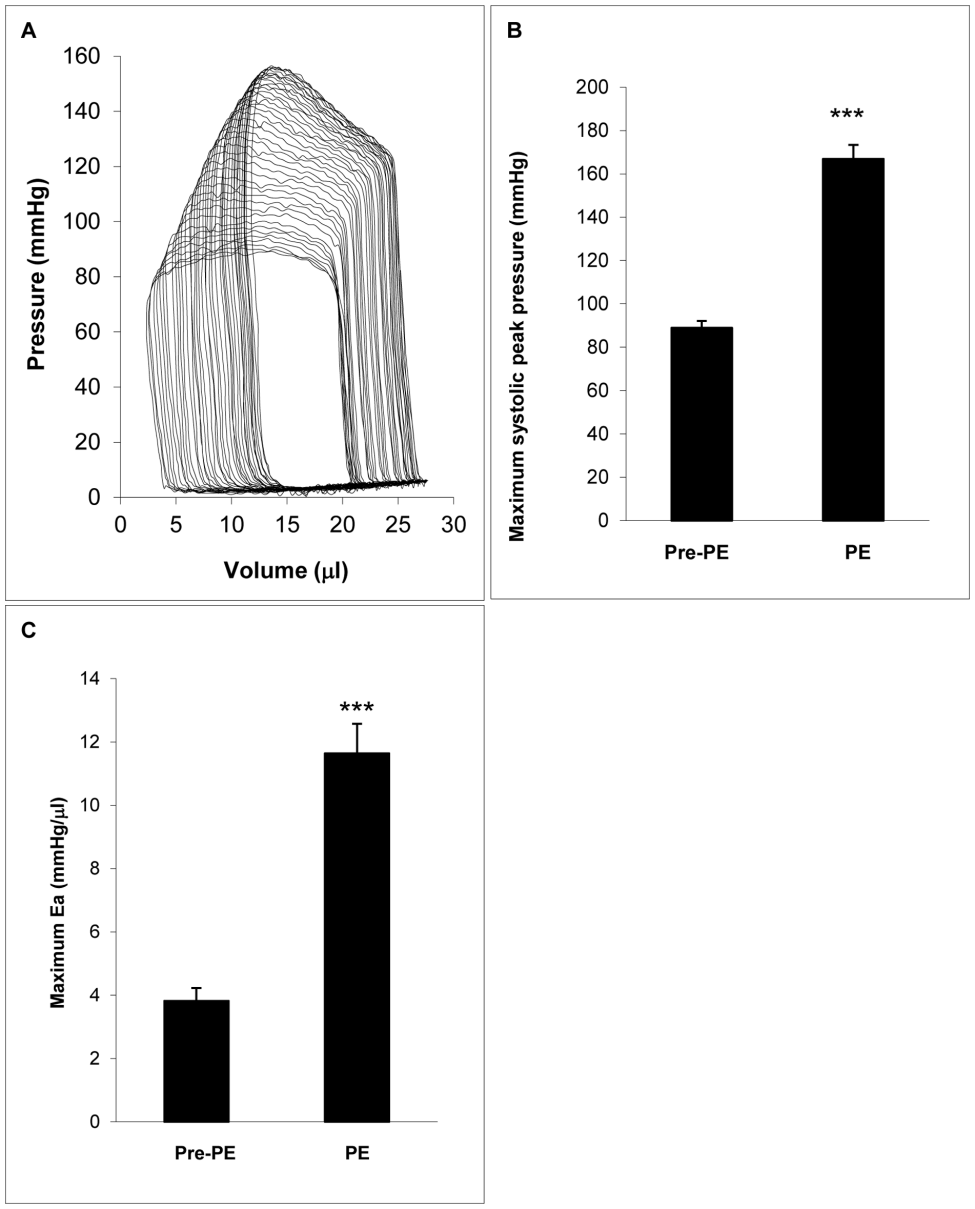
Arterial load was acutely increased by phenylephrine intravenous administration. A: Real time recording of series of pressure-volume loops during phenylephrine injection by Millar conductance catheter. The mean systolic peak pressure (B) and the mean arterial load (Ea) (C) of 10 mice were calculated before phenylephrine injection (Pre-PE) and at the maximum effects of phenylephrine (PE). PE = phenylephrine, Ea (effective arterial elastance) = Pes/SV (end systolic pressure/stroke volume).

The administration of phenylephrine produced a transient and significant increase of ventricular afterload characterized by systolic peak pressure and arterial load characterized by Ea (Figure 1). The maximum systolic peak pressure of LV increased 87.6% and the Ea increased 207%. The effects of phenylephrine reached the maximum within an average of 4 seconds after intravenous injection and then started to fade.

### τ and Heart Rate (HR), End Diastolic Pressure (Ped), and Arterial Load (Ea)

As described above, we first conducted the multi-regression of tau (including τ_Weiss_, τ_Glantz_, and τ_Logistic_) versus HR, Ped, Ea, and Ea^2^ for all mice in this study by using equation 4 (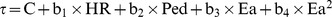). The results of the regression showed that only coefficients (b_3_ and b_4_) of Ea and Ea^2^ were significant (p<0.05) in every mouse and the coefficients (b_1_ and b_2_) of HR and Ped were non-significant (p>0.05). These results suggested that τ was dependent on the arterial load (Ea) and independent on heart rate and end diastolic pressure in this study. The biphasic linear regression analysis by using equation 5 (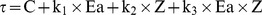) showed the coefficient k_3_ was statistically significant in every mouse (p<0.001). The significance of k_3_ suggested the existence of the inflection point. Compared to the single linear regression, the biphasic linear regression had a significantly greater R^2^ with p<0.001 on the F-test in every mouse. (See Appendix S1 for details regarding sample analysis).

### Inflection Points

**Figure 2:**
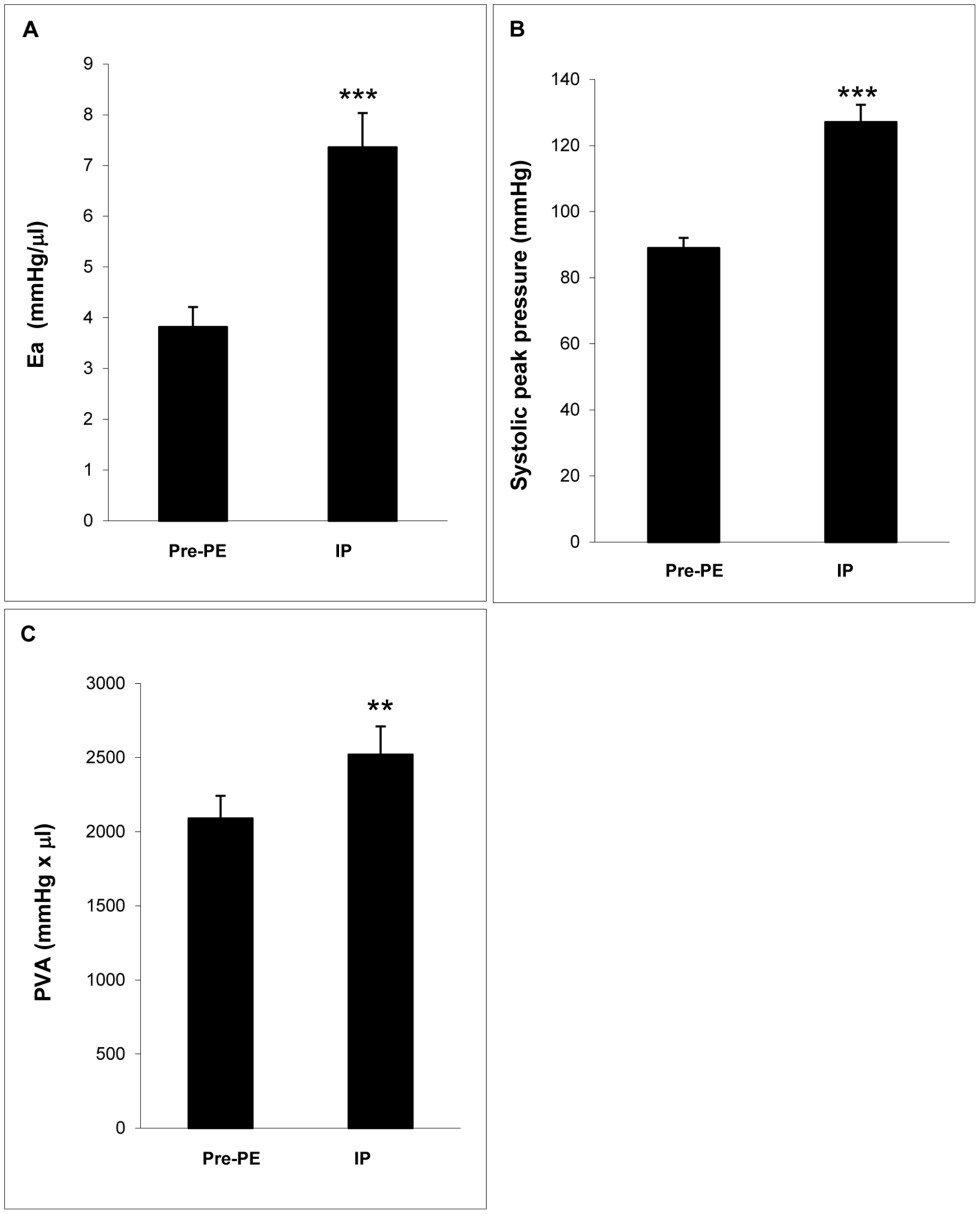
The Ea (A), PVA (B), and systolic peak pressure (C) at inflection point. The Ea, PVA, and systolic peak pressure were significantly greater at inflection point than those at baseline. PE: phenylephrine; IP: inflection point. **: p<0.01, ***: p<0.001, self-paired t-test.

**Figure 3:**
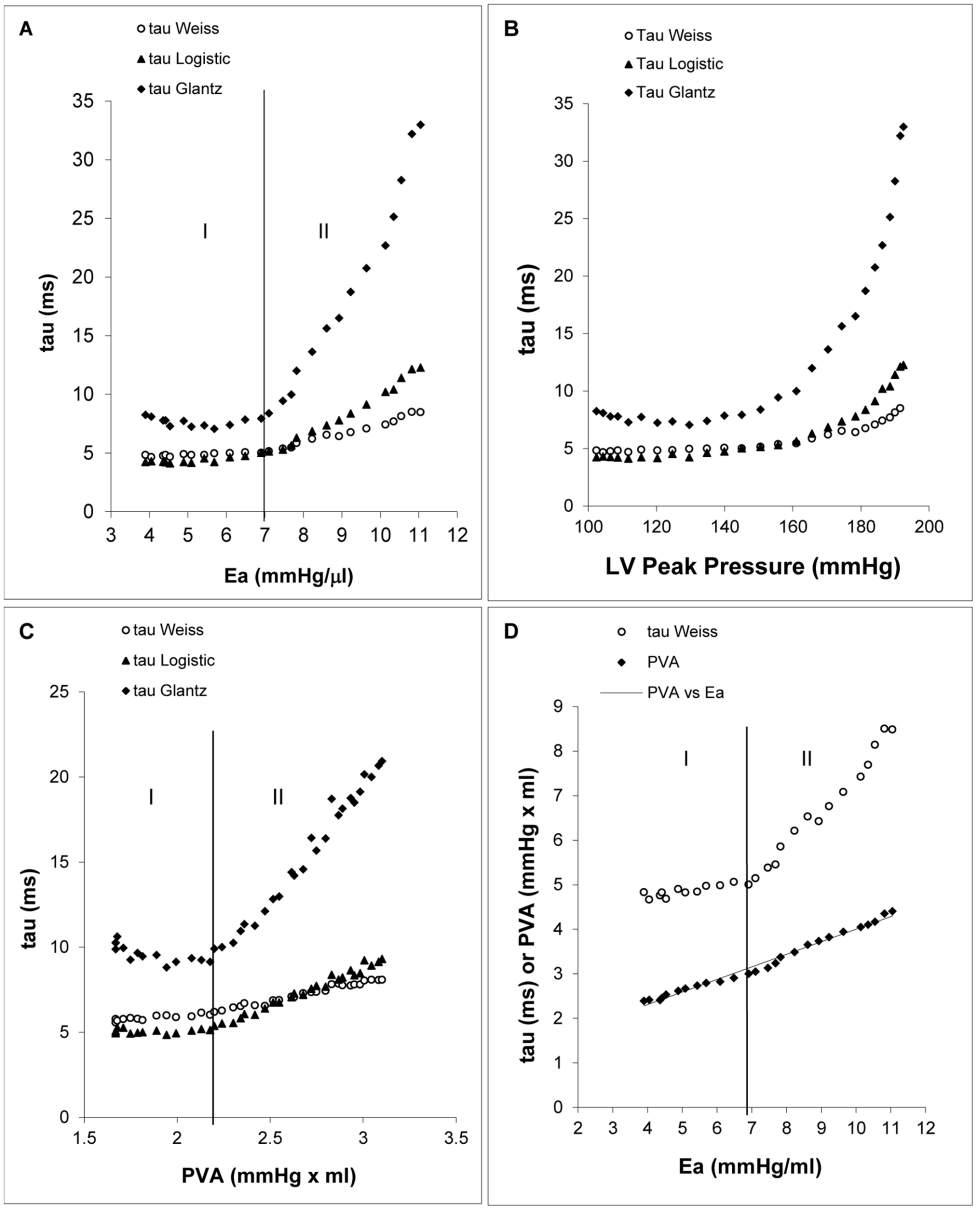
The relationship between τ, Ea, LV peak systolic pressure, and pressure-volume area (PVA). Plots of the three τ computations versus Ea (A), peak systolic pressure (B), and PVA (C) with phenylephrine injection. (D) Combined plots of τ (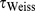) and PVA versus Ea (PVA = 282.63×Ea +1178.5, R^2^ = 0.9858).

The inflection point was defined as the end of phase I and the beginning of phase II in the plot of τ versus Ea or PVA. (See Appendix S1 for more detailed explanation of the calculation of inflection point). The existence of the inflection point was proved mathematically with biphasic linear regression. According to our data, the inflection point was at Ea = 7.36±0.68 mmHg/µl, the systolic peak pressure = 127±5 mmHg, and PVA = 2521±190 mmHg*µl across the animals (Figures 2A, B, and C). Graphic analysis showed the inflection point to be the same across all three τ’s (Figure 3A). Compared with those prior to phenylepherine injection, at the inflection point Ea increased 92.9%, systolic peak pressure increased 42.9%, and PVA increased 20.6% (p<0.01 or 0.001).

### τ and Ea

After we found the biphasic linear regression to be a better mathematical model for the relationship between τ and Ea, we applied the biphasic linear regression to every mouse. The induction of increased arterial load with phenylephrine uniformly revealed a biphasic characteristic using 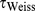, 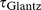, and 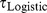 computations (Figure 3A). Tau versus Ea revealed a flat linear phase I and a significantly linear inclined phase II. Moreover, the biphasic linear regression of all τ calculations versus Ea demonstrated that the phase I slopes were not significantly different from zero. However, in phase II there was a positive slope (Figure 3A and Table 1). Additionally, in phase II as Ea increased, 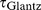 increased the most, 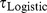 increased less, and 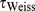 increased the least (p<0.01) (Figure 3A and Table 1). The plot of τ versus LV peak systolic pressure showed a curvilinear curve rather than a biphasic linear curve (Figure 3B).

**Table 1:** Coefficients from regression of τ versus Ea or PVA (n = 10).

	Phase I	Phase II
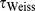 - Ea	0.09±0.04	0.59±0.09a, c,
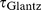 - Ea	−0.34±0.22	3.18±0.68a, b, d
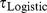 - Ea	0.045±0.07	1.04±0.20a, c, e
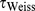 - PVA	−0.01±0.53	2.28±0.33a, c,
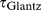 - PVA	−1.12±1.04	10.9±2.68a, c, d
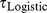 - PVA	0.32±0.46	3.62±0.78a, c, e

a p<0.01 versus slope = 0.

b p<0.05, c: p<0.01 versus phase I.

d p<0.01, 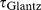 versus 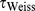 or 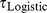 in phase II in the regression against Ea or PVA respectively.

e p<0.01, 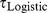 versus 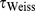 in phase II in the regression against Ea or PVA respectively.

### τ and PVA (Pressure-volume Area)

The plot of τ against PVA also showed a biphasic curve after phenylephrine administration (Figure 3C and Table 1). In phase I, regression-related coefficients of PVA versus 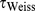, 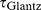, and 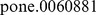 were not significantly different from zero. In phase II after the inflection point, the regression-related coefficients of the three τ measurements against PVA were significantly greater than zero. All coefficients in phase II increased by approximately 10 times for each mouse as compared with the coefficients in phase I. Similarly, the plot of τ versus PVA demonstrated greater slope for 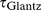 than 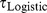 and 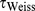.

### τ, PVA, and Ea

There was a linear relationship between PVA and Ea as the arterial load increased with phenylephrine injection in every mouse (p<0.01) (Figure 3D). The relationship of τ, PVA, and Ea was further clarified when those three variables were plotted in the same graph (Figure 3D). In phase I, as Ea increased, PVA increased in direct proportion, but τ did not change. In phase II, after the inflection point, as Ea increased, PVA was further increased linearly and subsequently τ began to increase (Figure 3D).

### τ at Baseline, Inflection Point, and Maximum Arterial Load

**Figure 4:**
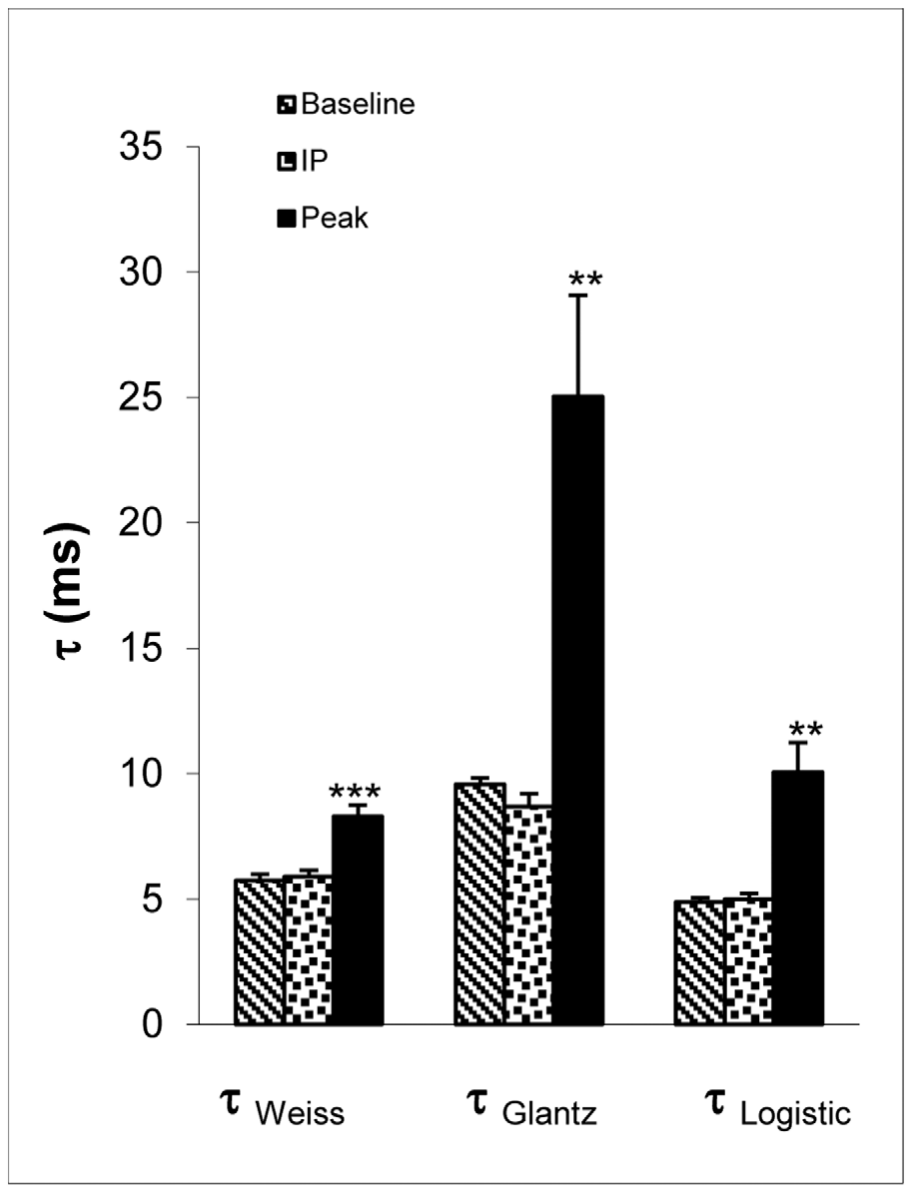
Tau at baseline, inflection point (IP), and at peak effect of phenylephrine. **: p<0.01; ***: p<0.001, self-paired t-test of black bar versus texture bar or dotted bar in the same category. IP: inflection point.

Three time points (baseline, inflection point, and maximum arterial load) were chosen as snapshots to further describe changes in τ during arterial load increase. At the inflection point, the arterial load characterized by Ea was almost doubled (Figure 2A), but there was no significant change in τ, including 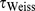, 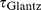, and 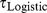 (p = 0.28, 0.15, 0.55 respectively; self-paired t-test) (Figure 4). When Ea was tripled as the arterial load reached the maximum (Figure 1C), 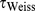 increased 44% (p<0.001), 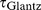 increased 162% (p<0.01), and 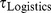 increased 107% (p<0.01) (Figure 4). Maximum 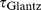 was greater than maximum 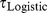 (p = 0.0022) which was greater than maximum 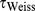 (p = 0.017) (Figure 4).

### Hemodynamic Changes with Phenylephrine Injection

**Figure 5:**
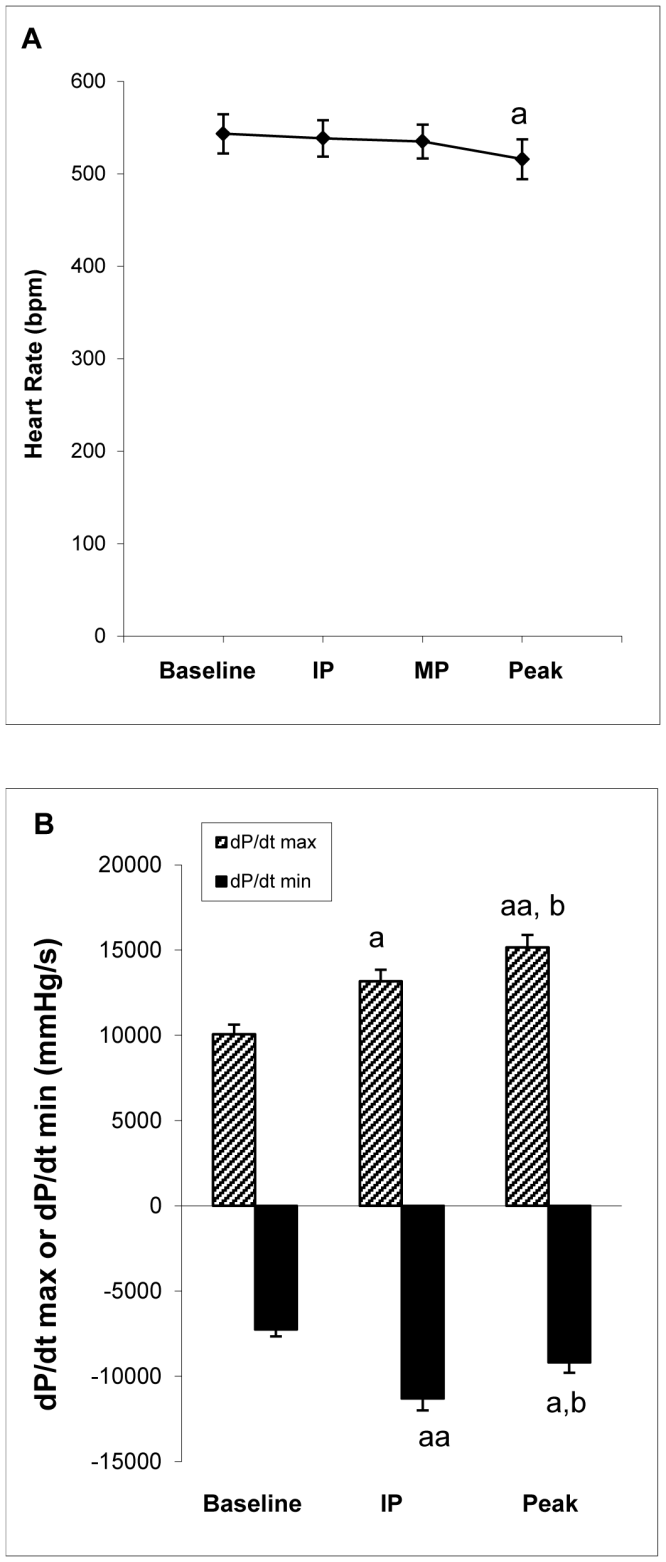
Changes in heart rate, dP/dt max, and dP/dt min with phenylephrine injection. (A): Heart rate before phenylephrine injection (baseline), at Inflection Point (IP), at middle point of phase II (MP), and at the maximum effects of phenylephrine (Peak). (B): dP/dt max and dP/dt min before phenylephrine injection (baseline), at inflection point (IP), and at the maximum effects of phenylephrine (Peak). a, aa: p<0.05 or p<0.01 versus baseline; b: p<0.05 versus IP. IP: inflection point; MP: middle point.

Compared to the heart rate at baseline, the heart rate did not significantly decrease at the inflection point and at the middle point of phase II (from inflection point to the maximum effect of phenylephrine) (p>0.05; self-paired t-test). At the maximum effect of phenylephrine, the heart rate decreased 5% (p = 0.046; self-paired t-test) (Figure 5A). The dP/dt max continuously increased as the arterial load increased. However, dP/dt min significantly increased at the inflection point, but decreased as the arterial load reached the maximum (Figure 5B).

## Discussion

This is the first study describing a biphasic linear relationship between τ (isovolumic relaxation time constant) and arterial load represented by Ea as investigated by recording a series of pressure volume loops in mice. The existence of the inflection point in this biphasic linear relationship was proved mathematically with biphasic linear regression. We proposed the concept of an inflection point, which is the connection of phases I and II. Before the inflection point (phase I), a small increase of arterial load and PVA (index of MVO_2_) had little effect on τ. After the inflection point (phase II), τ increased linearly as arterial load and PVA increased.

### Comparison with Previous Studies

Previously, a “J”-shaped pattern relationship of τ versus LV pressure was described by Leite-Moreira et al. in rabbits [27] and dogs [15]. In their study, 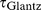 or 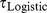 were computed to describe the LV pressure fall. The 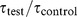 or τ was plotted against percentage of isovolumic pressure. In our study, we saw the same “J”-shaped pattern relationship when τ was plotted directly against LV peak systolic pressure (Figure 3B). However, we took advantage of the Pressure-Conductance Catheter System which measures the pressure and volume of LV at the same time. Ea was calculated from the pressure-volume relationship and used to represent the arterial load. The concept of Ea was originated by Sunagawa et al. [24], [25]. It is equal to end systolic pressure divided by stroke volume (Ea = Pes/SV), and also equal to total arterial resistance (R_t_) divided by the total period of heart beat (T) (Ea = R_t_/T). Ea is an index of arterial load and highly dependent on the arterial resistance [24], [25]. In our study, since the heart rate did not change throughout the effects of phenylephrine until the end of phase II, the total period of heart beat (T) did not change significantly during phenylephrine effects. Therefore, Ea directly reflected the total arterial resistance (R_t_). The plot of τ versus Ea demonstrated how arterial load/arterial resistance affects LV relaxation. Interestingly enough, the relationship was observed to be a biphasic linear relationship.

### Possible Reasons for Controversy

It has long been questioned whether τ is dependent on afterload, and there are several reasons for this doubt. First, most other studies ignore the intermediate phase, describing τ’s acute afterload response only at the baseline and endpoint, thus missing the biphasic properties that we observed. Second, some methods to induce afterload may not be adequate to cause a change in τ, which we found to be true in the mice with descending aorta occlusion. Third, the ascending aortic clamping may induce torsion of the heart’s position, especially in small animals as we saw in mice in our lab, which caused some artifacts. Fourth, the methods to compute τ (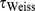, 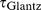, and 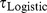) may also contribute to the controversy. Our results showed that 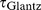 was much more sensitive to the change of afterload than 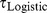 and 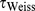 (Figure 3A and Table 1). Therefore, when different methods are used to compute τ, different results may be observed.

### Clinical Implications

Phenylephrine is an α adrenergic receptor agonist and favors α_1_ receptors over α_2_ receptors. It rapidly increases blood pressure by causing constriction of arterioles. Our results showed that a large dose of phenylephrine is toxic to the heart by causing diastolic dysfunction after the inflection point due to the increasing arterial load. It is consistent with the consensus that aggressively reducing arterial resistance may help diastolic heart failure patients. This study also provides a comparison for the studies of cardiac function in genetically-modified mouse models of cardiovascular disease, such as transgenic mice. It would be very interesting to see how τ responds to an increase of arterial load in SERCA 2a transgenic mice or phospholamban knockout mice. This research will help us to better understand the function of each individual protein related to the LV relaxation.

### Limitation

First, the criticism about pharmacologically induced increase of arterial load (such as with phenylephrine) is that it may alter heart rate and preload, thus skewing τ. In our study, the effect of phenylephrine was short (4 seconds) with no significant change in heart rate until the end of phase II. Only a 5% decrease in heart rate occurred at the maximum effect of phenylephrine. The multi-regression showed that neither heart rate nor end diastolic pressure had any significant effects on τ in the mice in this study. Ea was used to represent the arterial load and was described by Sunagawa et al. to be independent of preload [24], [25]. We changed preload in mice by compressing inferior vena cava or saline infusion, and we did not see significant changes of τ (data not shown). In the existing literature, studies also show that τ is relatively independent of preload [8], [11], [28]. Second, the insertion of the conductance catheter through the apex of the heart could negatively influence the function of the heart, and thus interpretations of the results are somewhat restricted to this type of procedure. Third, the mechanism of the biphasic change of tau as arterial resistance increases is unknown. Those models are not based on first principles but instead are just fits. More research is needed to investigate why the forms are what they are.

### Conclusions

We conclude that as afterload is increased acutely, τ changed in a biphasic pattern: a flat phase for 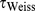 and 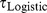 or a mildly decreasing phase for 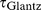, then a linearly increasing phase for all three τ’s. A similar biphasic pattern was observed in the plot of τ versus myocardial O_2_ consumption. The mechanism is unknown. Our hypothesis is that the biphasic pattern change of τ might be the consequence of decreased energy availability for relaxation due to increased myocardial O_2_ consumption for contraction as afterload increases acutely.

## Supporting Information

Figure S1 **Data analysis: a sample of biphasic linear regression.** The two empty circle data points ((6.63, 5.56) and (6.88, 5.38)) were in the hinge zone and used for the linear regressions of both phases I and II. The intersection point of these two linear regressions was calculated and the closest data point to the intersection point was defined as the inflection point.
(TIF)

Appendix S1 **A sample of data analysis.**
(DOCX)
