# Supplementary figures and images for: Biphasic Change of Tau (τ) in Mice as Arterial Load Acutely Increased with Phenylephrine Injection
Source: PLoS One. 2013 Apr 8;8(4):e60580. doi: 10.1371/journal.pone.0060580 (PMC3620408; doi:10.1371/journal.pone.0060580)

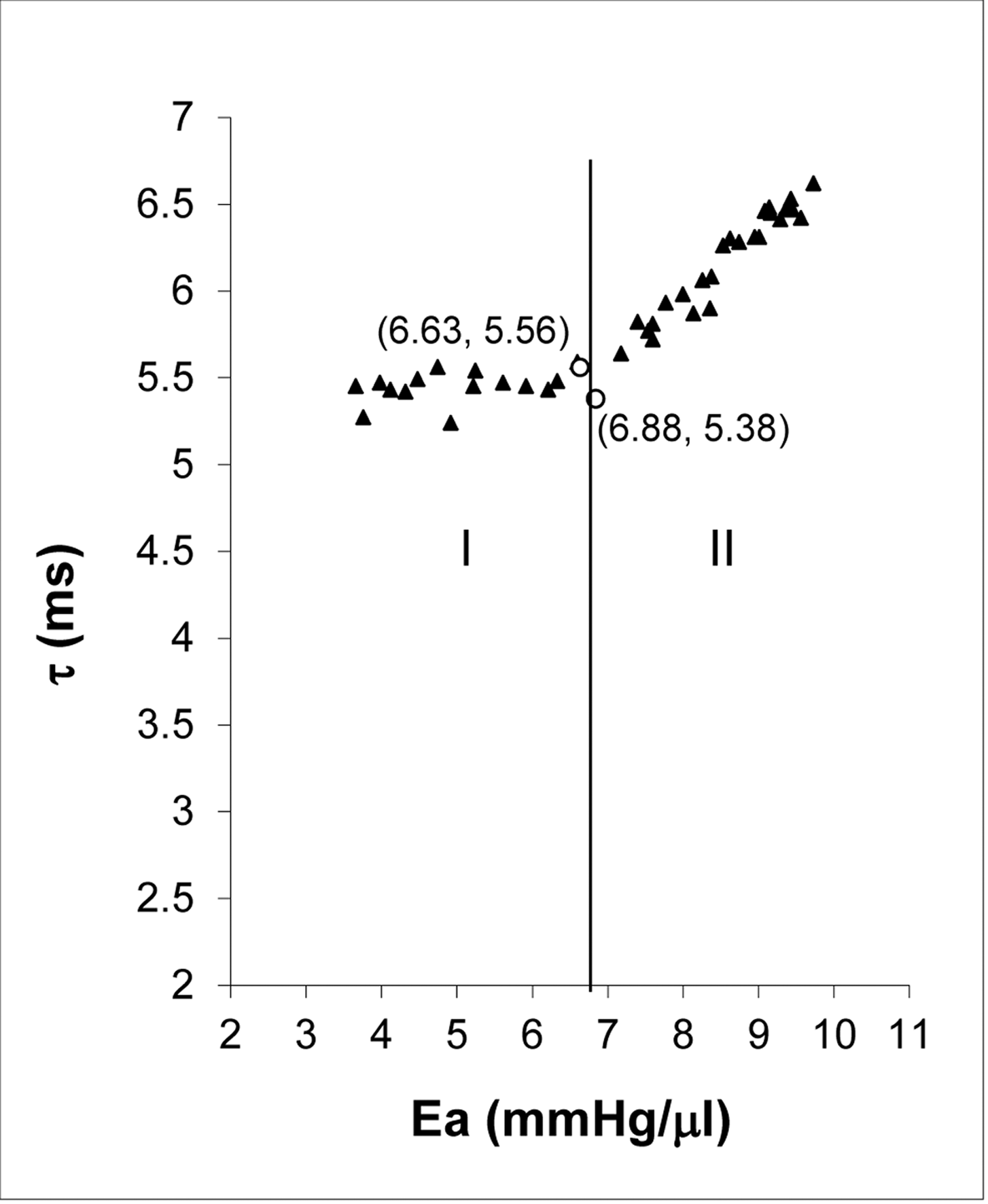

Supplement: Figure S1 — Data analysis: a sample of biphasic linear regression. The two empty circle data points ((6.63, 5.56) and (6.88, 5.38)) were in the hinge zone and used for the linear regressions of both phases I and II. The intersection point of these two linear regressions was calculated and the closest data point to the intersection point was defined as the inflection point. (TIF) [file pone.0060580.s001.tif]
