# Supplementary material for: Biphasic Change of Tau (τ) in Mice as Arterial Load Acutely Increased with Phenylephrine Injection
Source: PLoS One. 2013 Apr 8;8(4):e60580. doi: 10.1371/journal.pone.0060580 (PMC3620408; doi:10.1371/journal.pone.0060580)
Supplement: Appendix S1 — A sample of data analysis. (DOCX) [file pone.0060580.s002.docx]

**Appendix S1: A sample of data analysis:**

For this specific sample (Figure S1), graphical analysis suggested a non-linear relationship with an inflection point occurring somewhere in the region of 6.5 – 7. In order to set the inflection point, two separate linear regressions were conducted, one for all data points such that Ea < 7, and one such that Ea > 6.5. In other words, two of the data points were included in both regressions (see Figure S1, the two empty circle dots) to avoid deciding arbitrarily to which leg of the function each might belong. The first equation was τ_Weiss_ = 0.0284Ea + 5.30, R^2^ = 0.11. The second equation was τ_Weiss_ = 0.380Ea + 2.91, R^2^ = 0.94. By setting the two equations equal to each other, an intersection point of Ea = 6.79 was established. The closest point to the intersection point was chosen to be the inflection point. In this specific sample (Figure S1), Ea = 6.88 was chosen to be the inflection point, and phase I was defined as Ea < or = 6.88; phase II was defined as Ea > 6.88. The data of all the points was applied to piece-wise linear regression using equation 5 described in the data analysis. The above regression showed τ_Weiss_ = 5.30 + 0.0284 Ea - 2.38 z + 0.351 Ea*z, r^2^ = 0.974, where the slope of phase I is 0.0284 and the slope of phase II is 0.3794. The fact that the p-value was less than 0.0001 for k_3_ (k_3_ = 0.351) also confirmed the biphasic model for the plot of τ versus Ea. A linear regression of τ_Weiss_ on Ea was compared with a piece-wise linear regression, in which an interaction term (see equation 5) was constructed to allow the relationship between τ_Weiss_ and Ea to vary as function of two separate but joined lines, with an inflection point of Ea = 6.88. An F-test for the nested models showed that the piece-wise model fit statistically significantly better than a single linear model (p < 0.0001). The biphasic regression of τ versus PVA was carried out with the same method.
